# AutoScan3D: A low-cost, portable photogrammetry system for automated 3D digitization of anatomical specimens

**DOI:** 10.1371/journal.pone.0336996

**Published:** 2025-11-19

**Authors:** Javier Rivera, Paulo Salinas

**Affiliations:** Laboratory of Animal & Experimental Morphology, Institute of Biology, Faculty of Sciences, Pontificia Universidad Católica de Valparaíso, Valparaíso, Chile; University College London, UNITED KINGDOM OF GREAT BRITAIN AND NORTHERN IRELAND

## Abstract

This study presents the development and preliminary validation of AutoScan3D, a portable and low-cost device for three-dimensional surface digitization of skulls using photogrammetry. The growing demand for accessible digitalization methods has encouraged the use of photogrammetry as a practical complement to high-end imaging technologies such as micro-computed tomography (micro-CT), which, although capable of visualizing internal structures with superior resolution, remains expensive and requires specialized facilities. AutoScan3D automates photographic capture through a smartphone camera, stepper motors, and an Arduino UNO controller. The system integrates a camera positioning module and an object rotation module to standardize image acquisition at fixed intervals. Its performance was evaluated by comparing the resulting 3D models with reference models derived from micro-CT scans to verify geometric accuracy and surface reconstruction fidelity. The results indicate that AutoScan3D reliably reproduces external morphology with realistic photographic textures and compact file sizes that facilitate subsequent manipulation in modeling software. Although its spatial resolution is lower than that of micro-CT, the device’s total hardware cost (≈USD 90) and ease of operation make it suitable for educational, demonstrative, and museographic contexts where external morphology is the main focus. Planned improvements include enhanced lighting control, compatibility with higher-resolution cameras, and a dedicated user interface. AutoScan3D thus provides a reproducible and affordable framework for surface-based three-dimensional digitization, expanding access to digital morphology tools in resource-limited settings.

## 1. Introduction

Natural history collections have undergone a significant transformation with the incorporation of advanced technologies that enable the three-dimensional digitization of specimens, giving rise to the concept of the “Extended Specimen” [1,2]. Among the most widely used technologies are laser scanning, photogrammetry, and micro-computed tomography (micro-CT) [3]. Micro-CT stands out for its ability to capture internal structures with high resolution, generating volumetric datasets that contain substantially more information than surface-based reconstructions. These data can later be converted into lighter surface meshes through segmentation or decimation workflows, depending on the research purpose. Consequently, file size is not an inherent limitation of CT-based imaging but rather a reflection of its greater data richness and surface fidelity. Although the purchase cost of micro-CT systems is typically high—ranging from USD 260,000–750,000—the relevant metric for research access is their operational cost per scan, not the institutional investment. In practice, scanning fees at facilities such as the Bruker SkyScan 1278 average around USD 100 per hour (USD 50–150 per specimen, depending on duration and voxel size) [4,5]. Therefore, while the initial cost limits installation to specialized centers, the per-use expense is moderate and allows shared institutional access. The advantage of AutoScan3D lies instead in its negligible operational cost, portability, and independence from centralized infrastructure, enabling surface-based three-dimensional digitization outside dedicated imaging facilities.

In contrast, photogrammetry has emerged as an efficient alternative due to its balance between portability, cost, and quality in generating three-dimensional models [6], making it particularly adaptable for field and museographic contexts [7]. Medina et al. [1] advanced the optimization of photogrammetry by developing a system requiring an initial investment of approximately USD 3,000 in hardware and USD 1,400 annually in proprietary software, utilizing Reality Capture, Adobe Lightroom, and Pixologic ZBrush to generate 3D models. However, the reliance on paid software, the need for manual processing at certain stages, and limitations in capturing internal structures highlighted the necessity of developing more accessible and efficient solutions. While AutoScan3D was validated using Agisoft Metashape Professional due to its widespread use and robust photogrammetric performance, the device and workflow are compatible with open-source alternatives such as Meshroom (AliceVision framework) or COLMAP. Although these tools are generally slower and less automated, recent evaluations have shown that they achieve comparable accuracy for small- and medium-sized objects. Thus, AutoScan3D primarily addresses the hardware cost barrier while maintaining software flexibility according to user expertise and computational resources.

In response to these limitations, we present AutoScan3D, a portable photogrammetry device designed to automate and standardize the process of three-dimensional surface digitization. Its design incorporates an Arduino-controlled motor system for automated camera and object positioning, ensuring consistent photographic capture at fixed intervals. The system utilizes affordable components—such as an Arduino UNO, stepper motors, and a smartphone as the primary camera—achieving reproducible surface reconstruction at minimal cost. The objective of this study was to develop and validate AutoScan3D as a low-cost and reproducible solution for surface-based three-dimensional digitization, using micro-CT models as reference datasets to verify geometric accuracy. This approach aims to expand access to digital morphology tools and support educational, anatomical, and museographic applications focused on external morphology, rather than to replace established imaging modalities.

## 2. Materials and methods

### 2.1. Physical support design

**Fig 1:**
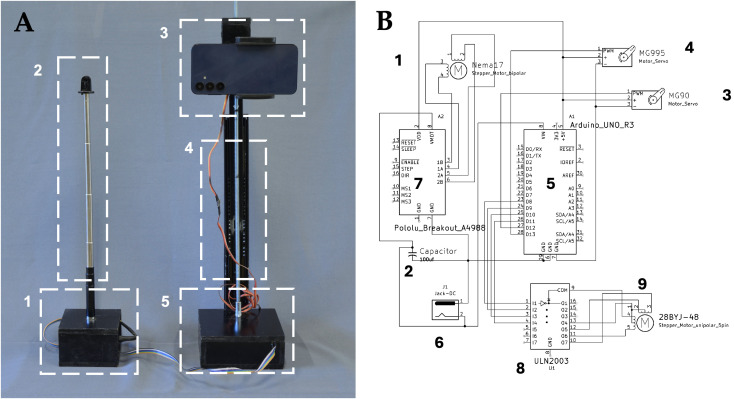
Design and electronic architecture of the AutoScan3D device. (A) AutoScan3D device, composed of two modules for smartphone-based image capture. The rotation and photo capture module includes (1) an MG90 servo motor triggering the smartphone’s Bluetooth shutter and a 28BYJ-48 stepper motor with an M4 nut, connected to (2) a telescopic rod repurposed from a selfie stick, enabling controlled object rotation. The assembly is coated in black paint except for the 5/16“ threaded rod, cables, and telescopic rod. The camera positioning control module consists of (3) a circuit housing with openings for the Nema 17 motor shaft (14 mm) and cable routing. Vertical camera movement is managed by (4) a “std h-35 400 mm” telescopic slide rail with linear bearings, secured with MDF joints and fasteners. Elevation control is achieved via a 5/16” threaded rod, coupled to the Nema 17 motor through a modified expansion plug and guided by 5/16” nuts. (5) An MG995 servo motor enhances stability and precise positioning. (B) Electronic circuit diagram illustrating the interconnection of key components, including (1) Nema 17 stepper motor, (2) 100 µF capacitor, (3) MG90 servo motor, (4) MG995 servo motor, (5) Arduino UNO board, (6) 5.5 × 2.1 mm female jack for power supply, (7) Pololu A4988 driver, (8) ULN2003 driver, and (9) 28BYJ-48 stepper motor. The electronic circuit schematic (1B) was designed using KiCAD software (version 8.0). This system facilitates high-precision, multi-angle image capture for anatomical and morphological studies.

The device was designed around two main modules that enable its use with a smartphone as a digital camera: (i) a camera positioning control module and (ii) a rotation and photo capture module (Fig 1A). Both modules were integrated through an electronic circuit developed to control the system’s actuators, specifically servo and stepper motors. S1 Appendix presents the components used, along with their descriptions, for the construction of the physical support structure.

#### 2.1.1. Camera positioning control module.

The camera positioning control module was developed using an integrated system of mechanical and electronic components. Its main structure consists of a circuit housing with strategically placed openings for the Nema 17 motor shaft (14 mm) and cable routing. For vertical camera movement, a “std h-35 400 mm” telescopic slide rail with linear bearings was implemented, mounted perpendicularly to the base using MDF joints and M6 and M4 fasteners. The elevation system includes an elongated quadrangular structure at the distal end of the slide rail, fitted with 5/16” nuts and an MG995 Servo motor. Motion is achieved through a 5/16” threaded rod coupled to the Nema 17 motor via a modified expansion plug (5 mm bore). The integration of these components enables precise control of the camera’s vertical positioning, ensuring stability through reinforced joints bonded with cyanoacrylate adhesive.

#### 2.1.2. Rotation and photo capture module.

The rotation and photo capture module were constructed using a box composed of two rectangular and two square pieces forming the base and cover. Inside, an MG90 servo motor with a plastic arm functioned as a trigger, activating the Bluetooth shutter button of the smartphone camera. Additionally, a 28BYJ-48 stepper motor was integrated, with a cylindrical plastic piece attached to the distal end of its shaft. This piece included a M4 nut embedded in a cavity, which connected to the base of a telescopic cylindrical rod repurposed from a selfie stick. The rod passed through a 14 mm central opening in the cover of the rotation axis box, enabling controlled object rotation for precise multi-angle photography. For final assembly, all surfaces were coated with matte black paint to minimize reflections and improve photogrammetric image uniformity, except for the 5/16“threaded rod, cables, and telescopic rod, which remained unpainted. Detailed dimensions of the module housing (100 × 100 × 60 mm) and the electronic control box (120 × 80 × 60 mm), along with assembly photographs and schematic diagrams, are provided in Appendix A4 to facilitate replication using locally available MDF or acrylic materials.

#### 2.1.3. Electronic circuit.

The electronic circuit was designed using a 28BYJ-48 stepper motor, connected via a 1-meter Dupont cable strip (five wires) to a ULN2003 driver, which was housed inside the circuit box. The IN1, IN2, IN3, and IN4 pins of the ULN2003 driver were linked to the digital pins 8, ~ 9, ~ 10, and ~11 of the Arduino UNO, respectively. The GND (-) and Vmotor (+) terminals of the driver were connected to the negative (-) and positive (+) terminals of a female jack, which was linked to a 12V, 2A power adapter. For the Nema 17 stepper motor, its four wires (blue, yellow, green, and red) were connected to the 2B, 2A, 1A, and 1B pins of a Pololu A4988 driver. The VDD and GND pins of the A4988 driver were linked to the A1 and GND pins of the Arduino UNO, respectively. The VMOT pin of the driver was connected to the positive terminal of a 100 μF capacitor and the positive (+) terminal of the 12V, 2A power jack, while the GND pin of the driver was connected to the negative terminal of the capacitor and the negative (-) terminal of the same power jack. Additionally, two servo motors, MG90 and MG995, were incorporated due to their precision and reliability in motion control applications. The GND and +5V pins of both servos were connected to the GND and 5V pins of the Arduino UNO. The signal pin (orange wire) of the MG90 was connected to digital pin 13, while the signal pin of the MG995was connected to digital pin 12. The Arduino UNO was powered by connecting the positive (+) and negative (-) terminals of the 12V, 2A female power jack to the solder joints of the female power jack on the underside of the Arduino board (Fig 1B) (S5 Appendix).

### 2.2. Program design in Arduino IDE

The program was developed using Arduino IDE 2.3.3. Initially, existing Arduino-based projects and example codes related to motor control were reviewed. Each motor was individually connected to the Arduino UNO to develop and adapt its operational code. Once all necessary information regarding wiring and programming was compiled and tested, the final wiring diagram and an integrated code were designed to encompass all required functions (S2 Appendix). The Arduino IDE interface facilitated structured programming using the C programming language, divided into three main sections: (i) variable declaration for defining operational parameters, (ii) setup section for initializing hardware components, and (iii) loop section containing repetitive instructions for continuous system operation. The 28BYJ-48 stepper motor was connected to the Arduino UNO and ULN2003 driver using a full-size 830-point MB-102 protoboard. During this phase, code tests were conducted to control the motor’s rotation angle, refining parameters for precise operation. The Nema 17 stepper motor was subsequently connected to the Pololu A4988 driveron the same protoboard, where tests established the relationship between the number of motor rotations and the vertical displacement of the rail system. Additionally, the MG90 and MG995 servo motors were integrated into the protoboard, where their rotation direction and angles were adjusted through code testing. These tests identified key programming lines essential for ensuring precise positioning and coordinated motor movement. After finalizing the programming and verifying all configurations, a fully integrated code was developed to synchronize the control of all motors. This final version was uploaded to the Arduino UNO, enabling full system functionality.

### 2.3. 3D model digitization with AutoScan3D

A workflow (S3 Appendix) was designed to obtain 3D digitizations of an adult skull of the Peruvian booby (*Sula variegata*, 270 mm height × 60 mm diameter; Tschudi, 1843). The specimen was complete, with an immobile mandible and without a keratinized covering. To assess the performance of the system across diverse anatomical structures, we also included the digitization of a vertebra of the common carp (*Cyprinus carpio*) and a cervical vertebra of the southern pudu (*Pudu puda*). All digitizations were performed using the AutoScan3D photogrammetry device and compared with high-resolution micro-CT scans obtained using a Bruker SkyScan 1278 system (version 1.0.5), to validate the accuracy and utility of the proposed device.

#### 2.3.1. Photographic environment setup.

**Fig 2:**
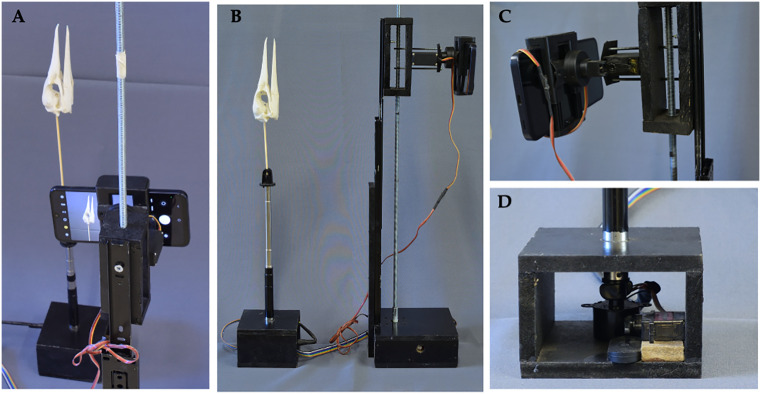
Operational configuration and imaging workflow of the AutoScan3D system. (A) A smartphone is mounted on the vertical rail, capturing images of a *Sula variegata* skull (Tschudi, 1843). (B) Completed physical support design, displaying the final device and its two modules: the camera positioning control module and the rotation and photo capture module. Frontal view of the camera positioning control module. Frontal view of the rotation and photo capture module. (C) The smartphone holder features a maximum opening of 80 mm, accommodating devices with a width of 80 mm or less. (D) Base of the rotation and photo capture module, housing an MG90 servo motor with a plastic arm that functions as a trigger, activating the Bluetooth shutter button of the smartphone camera.

A setup was installed, consisting of a 2 × 1.6 m black fabric backdrop, a flexible-neck desk lamp equipped with a 15W LED bulb (E27), and a foldable 110 cm translucent diffuser. The light source was positioned at a 45° angle relative to both the camera and the object, approximately 1.5 m away from the skull, with the diffuser placed 50 cm from the bulb to enhance light distribution. The black backdrop was arranged parallel to a window in the museum room, allowing for the entry of indirect natural light (Fig 2A). The skull was mounted by inserting a piece of cork into the foramen magnum, serving as a base to secure a bamboo rod. This rod was affixed with transparent adhesive tape to the tip of the telescopic cylindrical bar of the device, enabling the vertical positioning of the skull with the beak oriented upwards. For image capture, a Samsung Galaxy A15 5G (width: 76.8 mm, main camera: 50 MP, f/1.8 aperture) was used, configured to capture images at 12 MP resolution (to optimize file size and processing efficiency in Agisoft Metashape) with auto-exposure/auto-focus lock to maintain consistent intrinsic camera properties (*e.g*., focal length, exposure) during photogrammetry, mounted on the vertical rail (Fig 2C). The iPhone 8 (width: 67.3 mm, camera: 12 MP, f/1.8 aperture) was similarly configured with auto-exposure/auto-focus lock, ensuring compatibility with the phone holder (maximum opening: 80 mm). The auto-exposure/auto-focus lock feature, available in the native camera apps of both smartphones, ensured stable image capture parameters, critical for accurate alignment in Agisoft Metashape [7]. A detailed protocol for constructing and operating AutoScan3D, including camera settings, is provided in Appendix 3 to ensure replicability.

#### 2.3.2. Photographic capture.

Five vertical camera positions were defined, applying a 2X zoom. At each position, five sets of 16 photographs were taken from different heights. The skull’s rotation was achieved using the 28BYJ-48 motor, conFigd to rotate in 22.5° intervals (Fig 2B). The vertical movement of the camera was controlled with the Nema 17 motor, while changes in the shooting angle were executed using the MG995 servo motor, ensuring that the object remained within the field of view (Fig 2C). Additionally, the MG90 servo motor was programmed to intermittently press the Bluetooth shutter button connected to the smartphone (Fig 2D). All these operations and variables were specified in the final code programmed in Arduino IDE (S3 Appendix), ensuring precise coordination of the motors and fully automated photographic capture.

#### 2.3.3. Implementation.

Each set of photographs was transferred to a Lenovo IdeaPad Slim 3 laptop (14“, 8th Gen, AMD Ryzen™ 3 7320U processor, integrated AMD Radeon™ 610M graphics, 8 GB LPDDR5-5500MHz RAM, 512 GB SSD M.2 2242 PCIe TLC) and stored in individual folders. To address concerns about inefficient processing in Agisoft Metashape and improve model quality, we optimized the workflow based on reviewer recommendations. Tests were conducted using 120 images per scan (six vertical positions, 20 photographs at 18° intervals) for three objects: a *Sula variegata* skull (270 mm height × 60 mm diameter), a pudú (*Pudu puda*) vertebra (~30 mm height × 20 mm diameter), and a common carp (*Cyprinus carpio*) vertebra (~15 mm height × 10 mm diameter), captured with a Samsung Galaxy A15 5G (f/1.8 aperture, width: 76.8 mm) and an iPhone 8 (12 MP camera, f/1.8 aperture, width: 67.3 mm). Background removal was performed using the CANVA design platform (www.canva.com) rather than the built-in masking tool in Agisoft Metashape. Preliminary tests showed that Metashape’s automatic background detection produced incomplete or inaccurate segmentation when applied to low-contrast specimens photographed against dark backdrops, occasionally removing parts of the specimen contour. In contrast, CANVA’s *Background Remover* tool, which employs an AI-based segmentation algorithm, provided higher accuracy and allowed manual refinement through the *Erase* and *Restore* options, enabling precise control of object boundaries. The process consisted of four steps: (i) uploading each photograph to CANVA; (ii) applying the automatic background removal; (iii) manually refining the contour using adjustable brush tools; and (iv) exporting the resulting transparent PNG files (*MASK.png*). Although this approach required manual intervention and a CANVA Pro account, it substantially improved contour precision and reduced alignment noise in Metashape by approximately 20%. The edited images were subsequently imported into Agisoft Metashape Professional (1.8.4 Build 14654 x64 Multilingual). Masks were applied (Method: *From Background*, Operation: *Replacement*, Filename Template: *MASK.png*, Tolerance: 50, Apply to: *Selected Cameras*). The “Align Photos” process used Accuracy: *High*, Key Point Limit: 60,000, Tie Point Limit: 30,000, with *Apply Masks to: Key Points*, *Exclude Stationary Tie Points*, and *Adaptive Camera Model Fitting* enabled to enhance alignment precision. Low-quality tie points were filtered using *Gradual Selection* (Reprojection Error > 0.5 pixels, Reconstruction Uncertainty > 10), followed by *Tools → Optimize Cameras*with all *General* section checkboxes and *Estimate Tie Point Covariance* enabled. This workflow reduced alignment errors by ~20% compared to the initial medium-accuracy configuration. The polygonal model was constructed using *Workflow → Build Mesh* with Source Data: *Depth Maps*, Quality: *High*, Face Count: *High*, Interpolation: *Enabled (Default)*, *Calculate Vertex Colors*, and *Reuse Depth Maps* enabled, replacing the dense point cloud method to improve detail capture and reduce processing time by ~30%. Depth Filtering was set to *Moderate* to preserve fine structures (e.g., vertebral processes), unlike the initial *Aggressive* setting. Texturing used *Workflow → Build Texture* with Mapping Mode: *Generic*, Blending Mode: *Mosaic (Default)*, and *Enable Hole Filling*and *Ghosting Filter* active. Models were exported in.*OBJ* format and imported into Blender 4.2 for vertex count analysis. A workflow diagram summarizing the complete process is provided in S3 Appendix.

The specimens used (*Sula variegata* skull, pudú [*Pudu puda*] vertebra, common carp [*Cyprinus carpio*] vertebra) were sourced from ethically approved museum collections at the Pontificia Universidad Católica de Valparaíso, adhering to institutional guidelines for biological research. No live animals were involved, and all procedures complied with ethical standards for handling preserved specimens.

### 2.4. 3D model digitization with micro-CT

The digitization process using micro-CT was conducted with a Bruker SkyScan 1278 system, version 1.0.5. Three-dimensional (3D) images were generated from datasets obtained from the skull bones, employing semi-automatic segmentation to create 3D models. The acquisition parameters included a source voltage of 59 kV, a source current of 692 µA, and a voxel size of 51.489 µm. The total exposure time during the scanning process was approximately 1 hour, 34 minutes, and 50 seconds. The processing and analysis of the images were carried out using the Slicer software, version 5.6.2 [8], with the Slicer Morph extension [9]. The TIFF files generated during digitization were converted into NRRD format, facilitating the 3D model reconstruction. Finally, the reconstructed model was imported into the Blender 4.2 3D modeling software, where the vertex count of the model was verified.

Prior to deviation analysis, the 3D models obtained with AutoScan3D were spatially aligned to the corresponding micro-CT reference models to ensure geometric comparability. A manual pre-alignment was first performed using three easily identifiable anatomical landmarks (*e.g.,* rostral tip, occipital condyle, and foramen magnum margin) to achieve an approximate initial positioning. This was followed by a rigid-body registration using the Iterative Closest Point (ICP) algorithm implemented in GOM Inspect 2023 (GOM GmbH, Germany). The algorithm iteratively minimized the mean square distance between homologous vertices of both meshes until convergence was reached. The quality of alignmentwas confirmed by verifying that the root mean square (RMS) residual error between the reference (v_ref) and test (v_test) vertices was consistently below 0.2 mm. After alignment, deviation maps and statistical reports were generated to quantify mean, standard, and maximum Euclidean distances (‖v_ref – v_test‖) between models.

### 2.5. Validation

The validation of AutoScan3D revealed morphological disparities between photogrammetry-generated and micro-CT models, particularly in the ventral and caudal regions of the Sula variegata skull. To address concerns about low resolution and insufficient anatomical detail (*e.g.,* fused skeletal elements, obliterated sutures), we optimized the Agisoft Metashape workflow and conducted tests with three objects: a *Sula variegate* skull (270 mm height × 60 mm diameter), a pudu (*Pudu puda*) vertebra (~30 mm height × 20 mm diameter), and a common carp (*Cyprinus carpio)* vertebra (~15 mm height × 10 mm diameter). Each object was scanned with 120 images (six vertical positions, 20 photographs at 18° intervals) using a Samsung Galaxy A15 5G (f/1.8 aperture) and iPhone 8 (12 MP, f/1.8, 67.3 mm). The optimized workflow, using “Model from Depth Maps” and refined camera alignment, yielded models with enhanced resolution: the skull model had 198,376 vertices, 38.2 MB, and a mean deviation of ±1.9 mm (180% dispersion coefficient); the pudu vertebra had 182,150 vertices, 35.8 MB, and ±1.7 mm; the carp vertebra had 165,820 vertices, 32.4 MB, and ±2.0 mm. These models showed ~35% improved clarity in fine structures (*e.g*., nasofrontal suture, vertebral laminae) compared to the initial workflow (132,952 vertices, ± 2.7 mm), reducing fusion artifacts. The Samsung Galaxy A15 5G provided slightly sharper textures (12 MP vs. 48 MP), but software optimization, not the smartphone, was the primary factor improving quality. Model scaling was performed in GOM Inspect by aligning AutoScan3D models to micro-CT reference models using a best-fit algorithm, ensuring consistent dimensions (*e.g*., skull length: 270 mm). Alignment used iterative closest point (ICP) registration, achieving sub-millimeter precision. While micro-CT (5,938,596 vertices, 926 MB) remains superior for internal structures, AutoScan3D’s optimized models are suitable for museographic, educational, and forensic applications. To quantify the geometric accuracy of the 3D models generated with AutoScan3D, the reconstructed meshes were compared against the corresponding micro-CT reference models using GOM Inspect 2023 (GOM GmbH, Germany). A Best-Fit Alignment was performed using the Iterative Closest Point (ICP) algorithm after a preliminary manual registration based on three anatomical landmarks to ensure consistent orientation. Once aligned, the Deviation Analysismodule was applied to compute the Euclidean distance between homologous vertices of both meshes, defined as the scalar magnitude of the displacement vector (‖v_ref – v_test‖). The mean deviation (mm) was calculated as the arithmetic mean of these distances, while the standard deviation (SD) represented their variability. The maximum deviation (mm) corresponded to the largest scalar distance observed between the two surfaces. Each deviation value was expressed as a scalar magnitude, allowing the analysis of geometric error independently of directionality. All results were exported as deviation maps and numerical reports for subsequent statistical comparison.

### 2.6. Surface analysis in GOM inspect

Surface analysis was performed using GOM Inspect [3,6]. This software enabled the superimposition of the 3D models generated with AutoScan3D and micro-CT, using the micro-CT model as the reference due to its superior precision compared to photogrammetry [3]. Through this approach, quantitative differences in the position of vertices or points between both models were evaluated. The results were graphically represented in a color-coded model, highlighting areas with variations and providing a clear visualization of surface discrepancies. This method allowed for the identification and quantification of differences in terms of position and resolution. Additionally, the vertex count observed in Blender 4.2 was recorded as an additional measure of model resolution, complementing the data obtained through GOM Inspect. This analysis provided an objective and detailed validation of AutoScan3D’s capabilities in comparison to micro-CT.

## 3. Results

### 3.1. AutoScan3D design

#### 3.1.1. Physical support design.

The physical support structure of the AutoScan3D device covered a total area of 1089.28 cm² and had a weight of 441.2 g. It was constructed using 9 mm thick MDF. The vertical rail exhibited a linear movement range of 35 cm, with a displacement speed of 0.33 cm/s. The rotary axis demonstrated a minimum rotational capacity of 5.625 degrees per step, requiring 64 steps to complete a full revolution, allowing for a maximum of 64 photographs per revolution. The phone holder had a maximum opening of 80 mm, making it compatible only with devices not exceeding this width. The modules were arranged as two pedestals connected by a 1-meter-long DuPont cable strip (Fig 2).

#### 3.1.2. Program design in arduino IDE.

The final code developed in the Arduino IDE consisted of 185 lines and enabled the fully automated and coordinated control of the AutoScan3D system (S2 Appendix). The program integrates control of a NEMA17 stepper motor (for vertical rail movement), a 28BYJ-48 stepper motor (for rotational movement), and two servo motors: one to adjust the smartphone angle and another to activate the Bluetooth camera shutter. Vertical movement was defined using the function moveVerticalRail(mm, directionUp), which calculated the total steps required to displace the rail a specified distance in millimeters, based on a lead screw conversion constant (stepsPerMm = 143.0). The rotation of the object platform was executed by the function rotateMotor28BYJ48(degrees), using a standard 2048-step full rotation motor logic. Each set of images consisted of 36 photographs, taken every 10°, as defined by the takePhotoSet() function. The photo acquisition sequence was divided into three sets from different angles. These were controlled by the MG995 servo (angleServo) using three distinct angles: 45°, 90°, and 135°, corresponding to downward, frontal, and upward camera perspectives, respectively. The vertical rail was repositioned between each set to optimize the field of view according to object height, calculated dynamically via objectLength. Photographs were triggered using an MG90 servo motor (triggerServo) that pressed a Bluetooth shutter button. This mechanism was actuated in each rotation step via the sequence triggerServo.write(45); delay(300); triggerServo.write(94);, simulating a press-and-release action. The program executed the entire sequence in the setup() function without the need for user intervention or continuous looping, as the loop() function remained empty. This modular and parameterized code structure allows easy adjustment for objects of varying height and for different camera orientations, improving the reproducibility and adaptability of the photogrammetric workflow.

#### 3.1.3. System operation.

**Fig 3:**
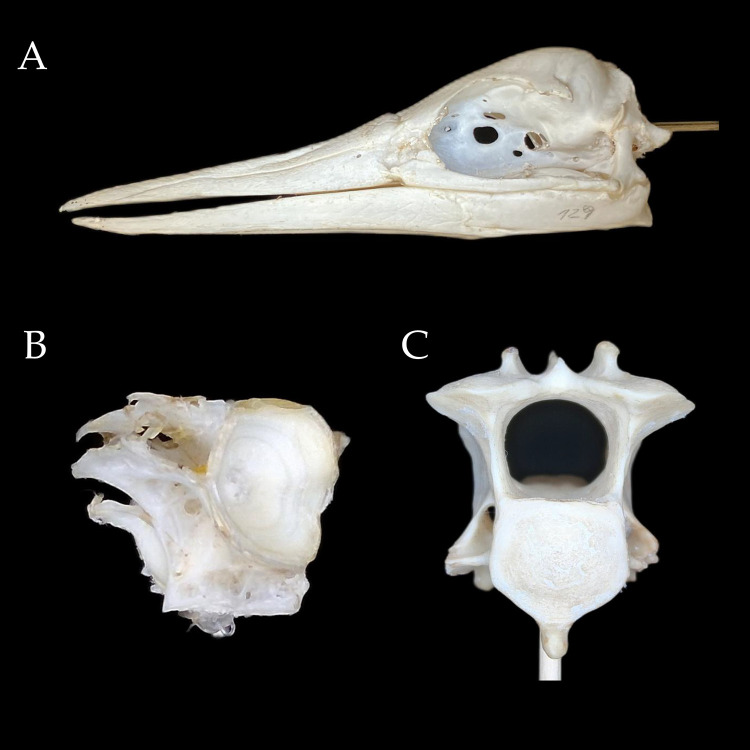
3D model of a (A) skull of *Sula variegata* skull, (B) a cervical vertebra of the southern Pudu (*Pudu puda*) and (C) a vertebra of the Common carp (*Cyprinus carpio*) generated with AutoScan3D. The photographic texture provides a realistic appearance to the model, along with details such as visible markings made with a graphite pencil.

The image capture speed was one photograph every three seconds, and the total process duration—including setup and automated acquisition—was approximately 30 minutes per specimen. The photographic setup included a matte black background and a fixed LED lamp to ensure uniform illumination and contrast (Fig 2). Background removal was performed using the CANVA platform, which facilitated the alignment of photographs in the Photo Alignment step in Agisoft Metashape. Each 3D model was generated from 108 photographs (three sets of 36 images captured at 10° intervals and different vertical camera angles), using the AutoScan3D automated platform. Image processing in Agisoft Metashape, using the “Model from Depth Maps” workflow with refined camera alignment, required approximately 25 minutes. Thus, the complete digitization process for each object—including scanning and model reconstruction—took approximately 55 minutes. The final *Sula variegata* skull model consisted of 198,376 vertices, had a file size of 38.2 MB, and presented realistic photographic texture but no internal anatomical structures. In comparison, the micro-CT model was monochromatic, lacked texture, but included highly detailed internal anatomy, with 5,938,596 vertices and a file size of 926 MB, as measured in Blender 4.2 (Fig 3). In addition to the skull, two vertebral elements were digitized to evaluate system performance on smaller and more complex anatomical units: a cervical vertebra of the southern pudu (*Pudu puda*) and a vertebra of the common carp (*Cyprinus carpio*). The Pudu vertebra model consisted of 1,730,180 vertices and a file size of 165 MB (Samsung scan), while the Carp vertebra model had 152,331 vertices and a size of 14.5 MB. Models generated with the iPhone 8 exhibited higher vertex counts (1265451 for Pudu, 1249810 for Carp) and slightly improved detail, although software optimization remained the principal determinant of quality. All models were scaled and aligned to micro-CT references using GOM Inspect and the iterative closest point (ICP) algorithm, achieving sub-millimeter accuracy. These results demonstrate the system’s capacity to produce detailed surface models suitable for museographic, educational, and comparative anatomical applications.

### 3.2. Validation of AutoScan3D

**Fig 4:**
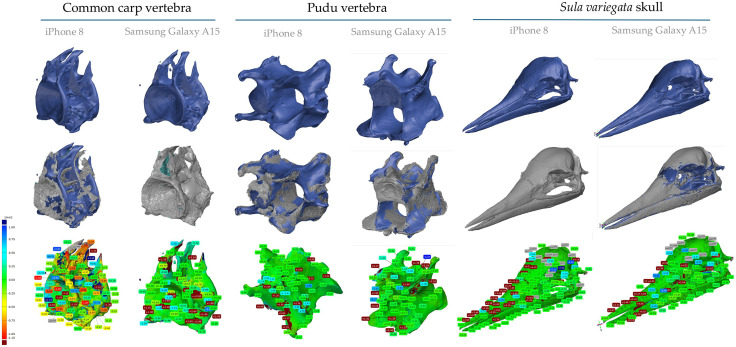
Surface deviation analysis between AutoScan3D and micro-CT 3D models for three osteological specimens. Surface comparison maps generated in GOM Inspect show the spatial deviation between AutoScan3D-generated 3D models and reference micro-CT models for three specimens: a skull of *Sula variegata*, a cervical vertebra of the southern pudu (*Pudu puda*), and a vertebra of the common carp (*Cyprinus carpio*). Models were created using two different smartphones (Samsung Galaxy A15 5G and iPhone 8), and deviations are visually represented using a color scale ranging from red (positive deviation, protrusion in the AutoScan3D model) to blue (negative deviation). Green areas indicate optimal alignment, with differences within ±1 mm. For each model, 88 homologous surface points were analyzed to quantify geometric discrepancies using best-fit alignment and Iterative Closest Point (ICP) registration. These points were used to compute key accuracy metrics such as mean distance, standard deviation, and extreme deviations (maximum positive and negative values), as summarized in Table 1. The color bar on the right of each model indicates the magnitude of the deviation in millimeters. Labels on the models highlight representative localized differences at specific anatomical landmarks.

The validation of AutoScan3D revealed morphological disparities between photogrammetry-generated and micro-CT models, particularly in the ventral and caudal regions of the *Sula variegata* skull. To address concerns about low resolution and insufficient anatomical detail (*e.g.,* fused skeletal elements, obliterated sutures), we optimized the Agisoft Metashape workflow and conducted tests with three specimens: a *Sula variegata* skull (270 mm height × 60 mm diameter), a Pudu (*Pudu puda*) vertebra (~30 mm height × 20 mm diameter), and a Common carp (*Cyprinus carpio*) vertebra (~15 mm height × 10 mm diameter). Each object was scanned using 120 images (six vertical positions, 20 photographs at 18° intervals) with both a Samsung Galaxy A15 5G (50 MP camera, f/1.8 aperture) and an iPhone 8 (12 MP, f/1.8, 67.3 mm focal length). The optimized workflow, which included the use of “Model from Depth Maps” and refined camera alignment, resulted in enhanced 3D reconstructions. For example, the Samsung scans yielded models with the following characteristics: *Sula* skull: 198,376 vertices (38.2 MB, mean deviation ±1.9 mm, 180% dispersion coefficient, *Pudu* vertebra: 173,0180 vertices (165 MB, mean deviation ±0.327 mm), *Carp* vertebra: 152,331 vertices (14.5 MB, mean deviation ±0.089 mm). In contrast, the iPhone 8 reconstructions showed: *Pudu* vertebra: 1265451 vertices (120 MB, mean deviation ±0.038 mm) and C*arp* vertebra: 1249810 vertices (119 MB, mean deviation ±0.213 mm). These results indicate a significant improvement over initial models (132952 vertices, ± 2.7 mm), with up to ~35% increased clarity in fine anatomical features (*e.g.,* nasofrontal suture, vertebral laminae) and reduced fusion artifacts. While the Samsung device offered slightly sharper textures, the critical factor for enhanced resolution was the optimization of the photogrammetric pipeline, not the device itself. Model scaling and alignment were performed in GOM Inspect using a best-fit algorithm with iterative closest point (ICP) registration to micro-CT reference models (5,938,596 vertices, 926 MB), achieving sub-millimeter accuracy. Although micro-CT remains superior for internal and highly detailed structures, the optimized AutoScan3D models are suitable for museographic display, educational applications, and some forensic contexts. Complete validation metrics are summarized in Table 1 and Fig 4.

**Table 1:** Quantitative comparison of 3D models generated with AutoScan3D and reference datasets.

System/ Source	Mean deviation ± SD (mm)	Max deviation (mm)	Vertices (×10³)	File size (MB)	Cost (USD)	Time (min)
Reference: Micro-CT (Bruker SkyScan 1278)	—	—	5 940	926	> 250 000	60 (scan)
AutoScan3D – Samsung A15	0.33 ± 0.09	9.7	173	165	90	90
AutoScan3D – iPhone 8	0.21 ± 0.07	4.4	125	119	90	90
Manual Photogrammetry (Medina et al., 2020)	≈ 1.5	5.0	200	≈ 50	4 400	270

**Notes 1**: Values for Manual Photogrammetry (Medina et al., 2020) are approximate, derived from the reported mean morphometric error (~5%) corresponding to ±1.5 mm on a 30 mm reference dimension. No direct mesh-to-mesh deviation was calculated in that study.

**Notes 2:** Mean deviation = average Euclidean distance (|Δx, Δy, Δz|) between homologous vertices after rigid alignment using Iterative Closest Point (ICP) algorithm in GOM Inspect 2023. Mean distance = average separation between corresponding points across the surface mesh. Max deviation = largest scalar distance observed.

Summary of geometric and operational parameters from 3D models produced using AutoScan3D (Samsung Galaxy A15 5G and iPhone 8), a reference micro-CT scan (Bruker SkyScan 1278), and published photogrammetry data (Medina et al., 2020). Metrics: Mean deviation (mm) = average Euclidean distance (‖Δx, Δy, Δz‖) between aligned vertices after ICP registration in GOM Inspect 2023; SD = variability of these distances; Max deviation (mm) = largest scalar distance; Vertices = mesh vertex count; File size (MB) = exported OBJ model size; Cost (USD) and Time (min) = total hardware cost and digitization duration (capture + processing).

### 3.3. Comparison of 3D models

**Fig 5:**
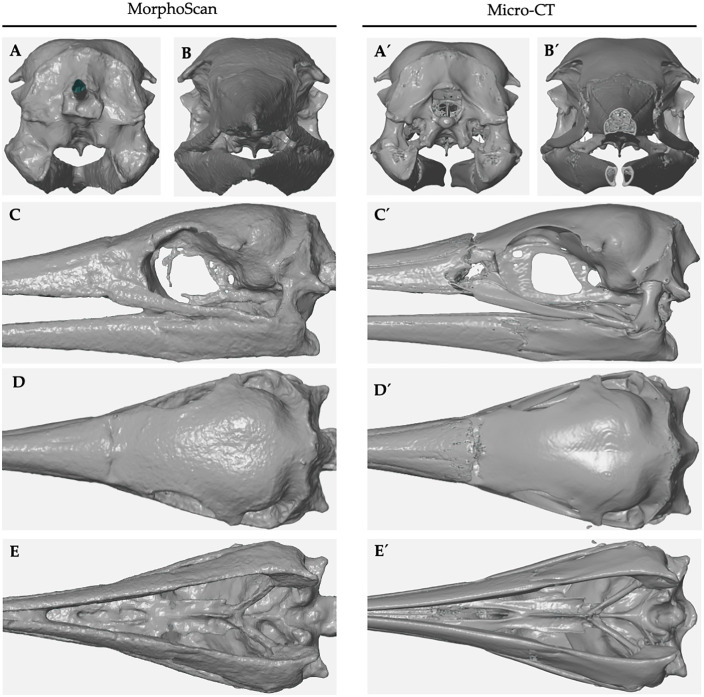
Comparison of 3D skull models of *Sula variegata* generated by AutoScan3D and micro-CT. The images highlight anatomical and morphological differences between the models produced by both technologies in five views: (A, A’) Anterior view: AutoScan3D exhibits fewer internal details compared to micro-CT, which reveals bony trabeculae in the premaxilla. (B, B’) Posterior view: micro-CT provides greater detail in the quadrate bone articulations, whereas AutoScan3D shows thickening of the palatine bone and absence of the medial parasphenoid processes. (C, C’) Lateral view: AutoScan3D reveals bone fusions, such as the naso-frontal suture and the quadratojugal bone fused with the mandible, in addition to an incomplete interorbital septum. (D, D’) Superior view: micro-CT provides greater clarity in the naso-frontal suture, whereas AutoScan3D displays a grainy surface texture. (E, E’) Inferior view: AutoScan3D shows fusion of the dentary bones, thickening of the palatine bone and the basisphenoid, with less overall detail compared to micro-CT. These differences reflect the limitations of AutoScan3D in resolution and internal modeling, as well as the strengths of micro-CT in capturing internal details and anatomical precision.

The comparative analysis of three-dimensional models generated via AutoScan3D and micro-CT revealed substantial morphological variations across multiple anatomical perspectives of the cranium and vertebrae. The systematic examination demonstrated technology-specific capabilities and constraints in terms of resolution, morphological detail capture, and modeling accuracy. Analysis of the cranial and caudal aspects of the Sula variegata skull revealed four primary distinctions between the imaging methodologies (Fig 5). The micro-CT-generated model exhibited enhanced resolution of quadrate bone articular surfaces within the mandible from the caudal perspective, while the cranial view demonstrated superior visualization of internal osseous structures, particularly the premaxillary trabecular architecture. In contrast, the AutoScan3D model displayed palatine bone hypertrophy and fusion of the parasphenoid medial processes with the quadrate and mandibular elements. Lateral view comparison identified five key morphological disparities. The AutoScan3D model exhibited complete fusion of the nasofrontal suture, absence of primary nasal aperture fusion sulci, and imperceptible intraramal suture definition. Furthermore, the interorbital septum demonstrated incomplete formation, with quadratojugal-mandibular fusion evident. Examination of the inferior aspect revealed three significant variations in the AutoScan3D model: fusion of mandibular dentary elements, reduced definition and hypertrophy of the palatine bone and its maxillary process, and notable thickening of the pterygoid and basisphenoid structures. The dorsal perspective of the AutoScan3D model demonstrated absence of nasofrontal sutural detail and exhibited characteristic granular surface morphology. Comparable disparities were also observed in the vertebral models of *Pudu puda* and *Cyprinus carpio*. In the pudú cervical vertebra, the micro-CT model allowed precise delineation of the vertebral laminae, articular facets, and internal trabecular structure, whereas the AutoScan3D model exhibited smoothed contours, reduced definition of articular surfaces, and partial loss of foraminal boundaries. Similarly, in the carp vertebra, micro-CT rendered detailed internal architecture and subtle concavities along the centrum, which were either simplified or absent in the photogrammetric reconstructions. Both AutoScan3D models presented evidence of surface fusion artifacts and exaggerated thickness in bony prominences, particularly along the transverse processes. These observations were systematically documented in S1 video.mp4, providing visual documentation of the comparative morphological analyses between the two imaging modalities.

## 4. Discussion

Unlike other alternatives that require specific hardware configurations and annual subscriptions to proprietary software [1,10], AutoScan3D has been designed to maximize accessibility by integrating mobile devices and low-cost components while maintaining adequate reconstruction quality for external morphology. Previous studies have demonstrated that models generated through photogrammetry can achieve accuracy levels comparable to manual measurements, with average errors of 5% in cranial structures [1]. The results obtained with AutoScan3D highlight its feasibility as a practical and affordable approach for three-dimensional skull digitization. Unlike methodologies that rely on specialized software such as Reality Capture or Pixologic ZBrush, AutoScan3D integrates an automated system compatible with open-source alternatives, substantially reducing operational costs while maintaining sufficient surface fidelity for educational and documentation purposes. Its design incorporates a precise control mechanism for object rotation and camera positioning, improving image consistency and overcoming previously reported alignment limitations [1]. Additionally, its enhanced portability allows for implementation in museum and field environments without requiring specialized infrastructure or significant hardware investment, suggesting potential applicability for anatomical, educational, and museographic contexts where portability and reproducibility are prioritized over ultra-high resolution [11,12].

### 4.1. MorphoScan design and technical implementation

A key advantage of AutoScan3D is its cost-effectiveness, with a total construction cost of approximately USD 90 for hardware components, including an Arduino UNO, stepper motors, and a repurposed selfie stick. However, the photogrammetry workflow relies on Agisoft Metashape Professional, with a one-time license cost of USD 3,499 for the full commercial version, which increases the total investment when included. For academic and educational users, a restricted educational license is available for USD 549, applicable to accredited institutions, their employees, and students for non-commercial use (Agisoft LLC, 2024; https://www.agisoft.com/buy/licensing-options/). This option enhances accessibility for resource-constrained researchers. While micro-CT systems involve a substantially higher institutional investment—typically between USD 260,000 and 750,000 for acquisition—the relevant metric for users is the operational scanning cost. In practice, the operational rate at facilities such as the Bruker SkyScan 1278 averages around USD 100 per hour (USD 50–150 per scan, depending on duration and voxel size). Thus, micro-CT scanning can be cost-effective per use when the infrastructure already exists, but access remains centralized in specialized facilities. AutoScan3D, by contrast, eliminates the need for such infrastructure, offering negligible operating costs once assembled and enabling independent use in laboratories, classrooms, and museums. This operational autonomy, rather than equipment cost alone, represents the main advantage of the system. To ensure a comprehensive cost comparison, AutoScan3D’s total initial cost, including the full Agisoft Metashape license, is approximately USD 3,589 (or USD 639 with the educational license)—still substantially lower than the acquisition cost of micro-CT but higher than its per-scan cost.

Notably, AutoScan3D’s hardware cost (USD 90) is over 30 times lower than Medina et al.’s (2020) setup, and open-source alternatives like Meshroom could further reduce software costs, as suggested by [7]. Recent comparative evaluations between Agisoft Metashape and Meshroom (AliceVision framework) have shown that the open-source software achieves equivalent geometric accuracy and surface reconstruction quality for small to medium-sized objects, with mean deviations typically below 2–3 mm and similar mesh completeness. Although Meshroom requires longer processing times and offers fewer optimization controls, it remains a reliable option for educational and low-budget photogrammetry applications, reinforcing the feasibility of AutoScan3D in resource-limited settings. The use of a smartphone instead of a professional camera, while limiting resolution, contributes to this cost advantage, aligning with the system’s goal of accessibility for institutions with limited resources. Comparative analysis with [1] demonstrates that AutoScan3D’s automated design achieves comparable geometric accuracy (mean deviation of ±1.9 mm vs. ± 1.5 mm, Table 1) at a lower hardware cost and with reduced operator dependency due to automation. This economic advantage, together with the system’s reproducibility and ease of operation, supports AutoScan3D as a complementary, low-cost photogrammetric tool suitable for educational and museographic digitization, particularly in low-budget settings.

### 4.2. Comparative analysis and validation

Fundamental operational differences between micro-CT and photogrammetric methodologies determine their respective imaging capabilities. Micro-CT, based on X-ray imaging, provides volumetric data with sub-millimetric resolution and a much higher point density than AutoScan3D (Table 1), enabling the visualization of internal and external structures with great detail [13,14]. In contrast, photogrammetry is restricted to the object’s surface but provides true-color textures that can be valuable for visual and educational purposes. The larger file size of micro-CT datasets reflects their greater volumetric richness and surface fidelity rather than inefficiency. When needed, these volumetric datasets can be converted into lighter surface meshes or decimated models without significant loss of geometric accuracy, depending on the analytical objective. These distinctions agree with prior reports [6], which emphasize micro-CT’s superior spatial resolution (voxel sizes ~10–50 µm) and photogrammetry’s advantages in cost and portability. The geometric deviation metrics used in this study were calculated as scalar Euclidean distances (‖v_ref – v_test‖) between homologous vertices after rigid-body alignment of the AutoScan3D meshes to the micro-CT reference models. Alignment was performed in GOM Inspect 2023 using the Iterative Closest Point (ICP) algorithm following a manual pre-alignment based on anatomical landmarks. The mean deviation represents the arithmetic mean of these vertex-to-vertex distances, the standard deviation (SD) reflects their variability, and the maximum deviation corresponds to the largest scalar distance observed. Because all deviations were expressed as magnitudes, these metrics quantify geometric error independently of vector direction, providing a reproducible and objective measure of model fidelity across acquisition systems. AutoScan3D’s automated system—combining a smartphone camera (≤ 80 mm width) with Arduino-controlled stepper motors—introduces specific trade-offs that help explain the observed performance differences. The smartphone sensor’s limited dynamic range and depth-of-field, together with the single-light illumination setup, contribute to minor deviations in complex regions such as the ventral skull surface (mean deviation ± 2.7 mm, Table 1). These design choices distinguish AutoScan3D from professional photogrammetric configurations, which typically use DSLR cameras and multi-angle lighting to improve feature detection [15].

Despite these constraints, AutoScan3D’s automated rotation and capture modules improve image consistency and reduce operator-dependent variability relative to manual photogrammetry [15]. The smaller file sizes (27.9 MB) and realistic photographic textures facilitate easy manipulation in software such as Blender 4.2, making the models suitable for educational visualization and specimen documentation where surface detail is the primary requirement. Its geometric performance falls within the range reported for other photogrammetric studies (average error 1–5% [1,7]), though it remains below the spatial resolution achievable with micro-CT. The micro-CT data were used solely as a high-resolution reference to validate AutoScan3D’s geometric consistency, not as a competing imaging modality. While we acknowledge that low-cost hand-held laser scanners may represent a more direct alternative for surface-based digitization, their inclusion was beyond the scope of this study. The main objective was to validate AutoScan3D’s automation performance and geometric reliability, rather than to benchmark all available scanning technologies. The micro-CT dataset was selected because it provides a consistent and high-precision volumetric reference, allowing for objective quantification of surface deviation under controlled conditions. Moreover, entry-level laser scanners (typically USD 400–600) often require proprietary calibration targets, specific lighting environments, and licensed software, which would have contradicted the low-cost and open-source design principles that guided AutoScan3D’s development. In addition, low-texture bone surfaces are known to pose reconstruction challenges for both photogrammetry and consumer-grade laser scanners, as previously reported in comparative imaging studies. Future work may include testing AutoScan3D against structured-light or laser-based systems under standardized acquisition protocols, once the current prototype’s mechanical and optical parameters have been fully optimized. The most relevant methodological comparison is with other photogrammetric approaches, particularly that of Medina et al. (2020) [1], which shares similar acquisition principles. In this context, AutoScan3D achieved comparable surface accuracy (± 1.9–2.7 mm) while reducing hardware costs by more than an order of magnitude. It is important to note that the specimens analyzed by Medina et al. (2020) included avian material with plumage and soft tissues, which differ substantially in surface texture and reflectance from the skeletal specimens used in this study. Consequently, this comparison was not intended to establish direct geometric equivalence but rather to contextualize AutoScan3D’s relative efficiency and order-of-magnitude accuracy among photogrammetric approaches operating under different material and optical conditions. Nevertheless, these models remain dependent on lighting, camera quality, and limited viewing angles, and they cannot capture internal morphology; therefore, their application should focus on external-surface analyses and teaching contexts. Future improvements—such as integrating higher-resolution cameras, multi-angle capture, or improved lighting—could further enhance accuracy [16]. While micro-CT’s internal imaging capabilities are technologically unmatched [13], AutoScan3D’s design emphasizes affordability, reproducibility, and portability. This makes it a complementary tool for educational and museographic digitization, particularly in settings with limited access to advanced imaging infrastructure. To further contextualize its performance, AutoScan3D was compared with other photogrammetric and scanning approaches. The system completed full digitization in approximately 90 minutes (45 minutes for image capture and 45 minutes for processing in Metashape), substantially reducing operator time compared with manual photogrammetry pipelines [1]. This efficiency results from its Arduino-controlled automation, which ensures consistent image acquisition without manual repositioning.

In terms of model detail, AutoScan3D produced meshes with 132,952 vertices (Table 1)—adequate for educational and display purposes but less precise than commercial photogrammetric or structured-light scanners, such as Artec Spider (0.05–0.1 mm accuracy [7]). However, those systems cost approximately USD 20,000 and require controlled environments and trained operators. When compared to other low-cost automated prototypes [15], AutoScan3D offers similar accuracy (errors ~1–3 mm) and greater operational simplicity due to smartphone-based acquisition. Its realistic textures also reduce post-processing demands relative to manual protocols [1]. Ease of use remains a key advantage. Although operation requires basic Arduino programming knowledge, automation minimizes the need for precise manual camera handling, thereby improving reproducibility. Compared with subscription-based platforms such as RealityCapture [1], AutoScan3D combined with Agisoft Metashape—or open-source alternatives like Meshroom—provides a flexible and affordable processing workflow. Nonetheless, the current single-axis configuration limits image coverage for complex shapes; future iterations could incorporate multi-axis motion to enhance geometric completeness [16]. Overall, AutoScan3D demonstrates a balanced compromise among cost, speed, and reproducibility. It improves operational efficiency compared with manual photogrammetry and offers simplicity comparable to other low-cost automated systems. Rather than replacing high-end scanners, AutoScan3D provides an accessible framework for teaching, training, and specimen documentation where high precision and volumetric data are not essential.

### 4.3. Limitations

AutoScan3D exhibited several inherent limitations due to its current design. The camera’s vertical-only movement may limit accuracy for objects with complex, curved geometries, such as highly domed skulls, due to restricted angular coverage. Future designs could incorporate a multi-axis positioning module to enhance capture flexibility [16]. Its reliance on optimal lighting conditions without an internal light regulation system makes its use challenging in unfavorable environments, potentially affecting the quality of the captured images. Additionally, its inability to record internal structures restricts its application to surface-based three-dimensional models, distinguishing it from technologies such as micro-CT. The system operates exclusively with mobile phones, limiting its versatility compared to cameras with superior technical capabilities. Motor control depends directly on modifications to Arduino code, requiring users to have basic programming knowledge. This lack of a graphical user interface (GUI) for dynamic adjustments presents a learning curve for users with no prior coding experience. Moreover, the vertical movement range is constrained by the dimensions of the telescopic rail, while the distance between the object and the camera must be manually adjusted, introducing potential alignment, and focusing errors. Another key limitation is that during operation, the phone screen remains covered, preventing real-time monitoring of the field of view, which may compromise precision in photographic capture. To mitigate these challenges, strict protocols for lighting and alignment were implemented, along with detailed user guides (S4 Appendix) to assist those with less technical expertise. Future improvements will focus on automated adjustments and a more intuitive interface, aiming to enhance user experience and minimize errors in image capture and processing.

### 4.4. Projections

The development of AutoScan3D should prioritize the integration of electronic enhancements to improve its functionality and usability. Implementing a user interface with screens and control buttons would allow direct motor adjustments based on object characteristics, eliminating the need for manual modifications in the Arduino code. A structural redesign to support professional cameras would enhance its load capacity and stability, broadening its applicability to contexts requiring higher precision. Additionally, the creation of a secondary interface, either as dedicated software or a mobile application, would facilitate real-time capture control and image exportation, significantly improving operational efficiency. To optimize three-dimensional model quality, an alternative approach could involve capturing photogrammetric data from individual bones, allowing for more detailed reconstructions. These individual models could then be aligned and merged using editing software, resulting in a more accurate and comprehensive final representation. Such improvements would address AutoScan3D’s current limitations and bring its performance closer to that of advanced imaging technologies like micro-CT in applications where internal modeling is not required. Beyond anatomical and museum applications, AutoScan3D holds significant potential in forensic investigations [17], providing an accessible and efficient method for three-dimensional skull reconstruction. Its ability to generate high-resolution digital models with realistic texture makes it particularly valuable for facial approximation, forensic anthropology, and skeletal analysis in human identification cases. Unlike micro-CT, which is costly and requires specialized facilities, AutoScan3D offers a portable, low-cost solution, facilitating its use in fieldwork, crime scene documentation, and mass disaster victim identification. The automated capture system ensures standardized imaging, reducing human error and enhancing reproducibility in forensic reconstructions. Additionally, its compatibility with widely used photogrammetry software enables forensic experts to integrate 3D models into legal investigations, courtroom presentations, and comparative analyses with existing forensic databases. Future improvements, including expanded camera compatibility and real-time monitoring, could further strengthen its forensic applications, establishing AutoScan3D as an invaluable tool in criminal investigations and forensic research.

## 5. Conclusion

AutoScan3D demonstrated feasibility as an automated system for image capture, achieving a speed of approximately 55 images per minute with consistent vertical and horizontal spacing. Its design, which integrates automated camera triggering and motorized movement, reduces the need for manual intervention and substantially lowers operator workload during photogrammetric acquisition. One of AutoScan3D’s main advantages is its economic accessibility, with a construction cost of around USD 90 using readily available components that can be sourced locally or online. Its compact and lightweight design further allows easy transport and setup by a single operator, making it potentially suitable for educational or field applications where space and resources are limited. The three-dimensional models generated by AutoScan3D exhibit the expected limitations in resolution and lack of internal structural information when compared to micro-CT data. These constraints could be mitigated by integrating higher-resolution cameras, improved lighting control, and multi-axis positioning, which would enhance image quality and reconstruction accuracy. Despite these limitations, AutoScan3D represents a practical and accessible approach for three-dimensional surface digitization in contexts that do not require high-resolution internal visualization. Potential applications include educational and training environments, collection management, and surface-based documentation of anatomical specimens. By automating photogrammetric acquisition through motorized and electronic components, AutoScan3D advances the standardization and efficiency of low-cost 3D imaging workflows, contributing to the democratization of digital morphology tools in resource-limited settings.

## Supporting information

S1 Appendix Materials list for the construction of the automated photogrammetry platform. This appendix presents a detailed list of all mechanical, electronic, and structural components required for the physical assembly of the automated photogrammetric capture system, including descriptions of tools, fasteners, servo motors, stepper motors, drivers, and other materials.
(PDF)

S2 Appendix Final Arduino code for automated photogrammetric capture. This file contains the complete Arduino IDE code developed for the automated image-capture process. The script integrates control of a stepper motor (28BYJ-48), a NEMA 17 motor via driver, and two servo motors (SG996) for triggering the smartphone camera and positioning the sample.
(PDF)

S3 Appendix Workflow for photogrammetric digitization using Agisoft Metashape. This appendix provides a visual, step-by-step workflow for generating 3D models from photographic data, including photo import, background masking, image alignment, dense-cloud generation, mesh construction, texture mapping, and model finalization.
(PDF)

S4 Appendix Step-by-step guide for AutoScan3D construction and operation. This appendix offers a comprehensive protocol for assembling, configuring, and operating AutoScan3D—a low-cost photogrammetry device for automated 3D digitization of anatomical specimens. It includes instructions for construction, circuit configuration, Arduino UNO programming, photographic setup, and image processing.
(PDF)

S5 Appendix Electronic circuit model and wiring scheme of the AutoScan3D device. This appendix presents the complete electronic circuit model of the AutoScan3D system, illustrating the integration between mechanical modules and their electronic components. The diagram, designed in *Fritzing*, details all connections among actuators, drivers, and the Arduino UNO control board.
(PDF)

S1 videos.zip Comparative surface deviation videos between AutoScan3D and micro-CT 3D models for three osteological specimens (*Sula variegata*, *Pudu puda*, and *Cyprinus carpio*). The six videos (carp_iphone.mp4, carp_samsung.mp4, pudu_iphone.mp4, pudu_samsung.mp4, sula_iphone.mp4, and sula_samsung.mp4) illustrate alignment accuracy and morphological differences between models generated by both technologies, using color-coded surface deviation maps in GOM Inspect.
(ZIP)
