# Supplementary material for: AutoScan3D: A low-cost, portable photogrammetry system for automated 3D digitization of anatomical specimens
Source: PLoS One. 2025 Nov 19;20(11):e0336996. doi: 10.1371/journal.pone.0336996 (PMC12629479; doi:10.1371/journal.pone.0336996)
Supplement: S1 Appendix — This appendix presents a detailed list of all mechanical, electronic, and structural components required for the physical assembly of the automated photogrammetric capture system, including descriptions of tools, fasteners, servo motors, stepper motors, drivers, and other materials. (PDF) [file pone.0336996.s001.pdf]

APPENDIX 1

Table 1. List of supplies for the design of the physical support.

SUPPLY	DESCRIPTION
2 X MDF cuts (x dimensions) 9mm	Structural material.
5/16″ Threaded rod	Long bolt connected to the stepper motor.
2 X 5/16″ Nuts	Fastening.
Cold glue	Adhesive for MDF.
10mm Drill	Creating holes in MDF.
Dremel-type Drill	
Telescopic slide std h-35 400 mm	Allows maintaining the vertical position of the camera.
2 x M3 x 75 mm bolts	Structural fastening.
2 x M3 nuts	
4 x M4 x 12 mm bolts	
2 x M4 x 25 mm bolts	
6 x M4 nuts	
3mm Wood drill bit	Creating holes in MDF.
4mm Wood drill bit	
14mm Wood hole saw drill bit	
5/16″ Anchor plug	Coupling between Nema17 and the 5/16″ threaded rod.
5mm Metal drill bit	Resizing the 5/16″ anchor plug.
Nema 17	Stepper motor for vertical control.
28BYJ-48	Stepper motor for angular photography control.
Arduino UNO	Development board.
Driver A4988	Communication driver between Nema 17 and Arduino UNO.
Driver ULN2003	Communication driver for 28BYJ-48.
100uF Capacitor	Necessary capacitor for the operation of Driver A4988 and Nema 17.
Servo MG90 + simple servo arm	Servo acting as a trigger for the Bluetooth shutter release for the cellphone.
Servo MG995 + simple servo arm	Servo controlling the vertical shooting angle of the cellphone and its camera.
Bluetooth shutter release for cellphone	Allows taking pictures remotely without a wired connection.
5.5x2.1mm 3-pin female jack	Connects the 12V 2A adapter to the circuit.
12V 2A Adapter	Powers the circuit with 12V 2A.
1m strip of 5 fused Dupont cables	Connects the 28BYJ-48 to the rest of the structure, improving system adaptability.
Assorted Dupont cables	Used for electrical connections between components.
PCB prototyping board 60 x 80 mm	Integrates the Arduino UNO board with other components, saving space and allowing wire connections without direct soldering.
1mm, 30g Solder	Used for electrical connections along with the soldering iron.
Assorted heat shrinks tubes	Protect electrical connections.
Single-row JST connector strips, male. Minimum 30 pins.	Soldered to the prototyping board for reversible connection to the Arduino UNO board.
Third-hand tool or helping magnifier	Aids in soldering by immobilizing components.
Cyanoacrylate glue 'Gotita'	Adhesive.
