# Supplementary material for: AutoScan3D: A low-cost, portable photogrammetry system for automated 3D digitization of anatomical specimens
Source: PLoS One. 2025 Nov 19;20(11):e0336996. doi: 10.1371/journal.pone.0336996 (PMC12629479; doi:10.1371/journal.pone.0336996)
Supplement: S2 Appendix — This file contains the complete Arduino IDE code developed for the automated image-capture process. The script integrates control of a stepper motor (28BYJ-48), a NEMA 17 motor via driver, and two servo motors (SG996) for triggering the smartphone camera and positioning the sample. (PDF) [file pone.0336996.s002.pdf]

S2 Appendix

Final code of the project developed in Arduino IDE

Final Arduino code for automated photogrammetric capture.
This file contains the complete Arduino IDE code developed for the automated capture of images in a photogrammetric setup. The script integrates the control of a stepper motor (28BYJ-48), a NEMA17 motor via a driver, and two servo motors (SG996) for triggering a smartphone camera and positioning the sample. The code includes parameters for step sequencing, timing, rotation steps, and synchronized image capture, enabling automated acquisition of 16 images per rotation cycle.

```
#include <Servo.h>

byte directionPin = 5;
byte stepPin = 4;

const float stepsPerMm = 143.0;  // steps per mm for the 5/16" UNC lead screw with NEMA17 motor

int pulseWidthMicros = 30;  // microseconds (not used)
int millisbetweenSteps = 2; // ms between steps
int IN1 = 8;  // 28BYJ-48 pins
int IN2 = 9;
int IN3 = 10;
int IN4 = 11;
int delayTime = 10; // delay ms per step for 28BYJ-48 motor

Servo angleServo;     // servo that moves the phone angle
Servo triggerServo;   // servo that presses the Bluetooth shutter

// Parameters:
float objectLength = 0;        // length of the object in mm
float baseDistance = 250;       // 25 cm = 250 mm constant distance from the base- to the center of the
photographed object

// Control of smartphone camera angle
const int angleDown = 45;
const int angleUp = 135;  // 45° up, adjust according to physical orientation

// Photo set
const int photosPerSet = 36;
const int degreesPerPhoto = 10;

// Function to move NEMA17 motor vertically in mm
void moveVerticalRail(float mm, bool directionUp) {
  digitalWrite(directionPin, directionUp ? HIGH : LOW);
  long steps = (long)(stepsPerMm * mm);
```

```
  for (long i = 0; i < steps; i++) {
    digitalWrite(stepPin, HIGH);
    delayMicroseconds(pulseWidthMicros);
    digitalWrite(stepPin, LOW);
    delay(millisbetweenSteps);
  }
}

// Function to rotate 28BYJ-48 motor by a specific angle (positive = clockwise)
void rotateMotor28BYJ48(int degrees) {
  int stepsPerRevolution = 2048; // steps per full rotation (360°)
  int stepsForRotation = (degrees * stepsPerRevolution) / 360;

  for (int i = 0; i < stepsForRotation; i++) {
    digitalWrite(IN1, HIGH);
    digitalWrite(IN2, LOW);
    digitalWrite(IN3, LOW);
    digitalWrite(IN4, LOW);
    delay(delayTime);
    digitalWrite(IN1, LOW);
    digitalWrite(IN2, HIGH);
    digitalWrite(IN3, LOW);
    digitalWrite(IN4, LOW);
    delay(delayTime);
    digitalWrite(IN1, LOW);
    digitalWrite(IN2, LOW);
    digitalWrite(IN3, HIGH);
    digitalWrite(IN4, LOW);
    delay(delayTime);
    digitalWrite(IN1, LOW);
    digitalWrite(IN2, LOW);
    digitalWrite(IN3, LOW);
    digitalWrite(IN4, HIGH);
    delay(delayTime);
  }
  // Turn off all pins to stop the motor
  digitalWrite(IN1, LOW);
  digitalWrite(IN2, LOW);
  digitalWrite(IN3, LOW);
  digitalWrite(IN4, LOW);
  delay(300);
}

// Function to take a photo set with object rotation
void takePhotoSet() {
```

```
  for (int i = 0; i < photosPerSet; i++) {
    rotateMotor28BYJ48(degreesPerPhoto); // rotate 10 degrees
    delay(200); // wait before triggering camera

    // Press Bluetooth shutter with small servo
    triggerServo.attach(13);
    triggerServo.write(45); // press
    delay(300);
    triggerServo.write(94); // release
    delay(1000);
  }
}

void setup() {
  Serial.begin(9600);

  pinMode(A0, OUTPUT);
  digitalWrite(A0, LOW);

  pinMode(13, OUTPUT); // Pin for shutter servo

  pinMode(IN1, OUTPUT);
  pinMode(IN2, OUTPUT);
  pinMode(IN3, OUTPUT);
  pinMode(IN4, OUTPUT);

  pinMode(directionPin, OUTPUT);
  pinMode(stepPin, OUTPUT);

  angleServo.attach(12);  // phone angle servo
  triggerServo.attach(13); // shutter servo

  // Suppose these variables are set before or via serial
  objectLength = 200; // example: 200mm (20 cm)

  // First action: move vertical rail up (half length + 100mm)
  float verticalMove = (objectLength / 2.0) + 100.0; // mm
  moveVerticalRail(verticalMove, true); // true = up

  // Set phone angle downward
  angleServo.write(angleDown);
  delay(500);

  // Take first set of 36 photos with rotation
  takePhotoSet();
```

```
  // Return 28BYJ-48 motor to initial position
  rotateMotor28BYJ48(-360); // rotate 360 degrees counterclockwise

  // Second front photo set (90° between phone and object)
  angleServo.write(90); // perpendicular angle
  delay(500);
  takePhotoSet();

  // Move vertical rail down (half length + 100mm)
  moveVerticalRail(verticalMove, false); // false = down

  // Set phone angle upward
  angleServo.write(angleUp);
  delay(500);

  // Third photo set with rotation
  takePhotoSet();

  // Return vertical rail to initial position (25 cm from base)
  moveVerticalRail(baseDistance - verticalMove, true); // move up if needed (adjust sign if needed)

  Serial.println("Sequence finished.");
}

void loop() {
  // Nothing to do in loop
}
```
