# Supplementary material for: AutoScan3D: A low-cost, portable photogrammetry system for automated 3D digitization of anatomical specimens
Source: PLoS One. 2025 Nov 19;20(11):e0336996. doi: 10.1371/journal.pone.0336996 (PMC12629479; doi:10.1371/journal.pone.0336996)
Supplement: S3 Appendix — This appendix provides a visual, step-by-step workflow for generating 3D models from photographic data, including photo import, background masking, image alignment, dense-cloud generation, mesh construction, texture mapping, and model finalization. (PDF) [file pone.0336996.s003.pdf]

APPENDIX 3

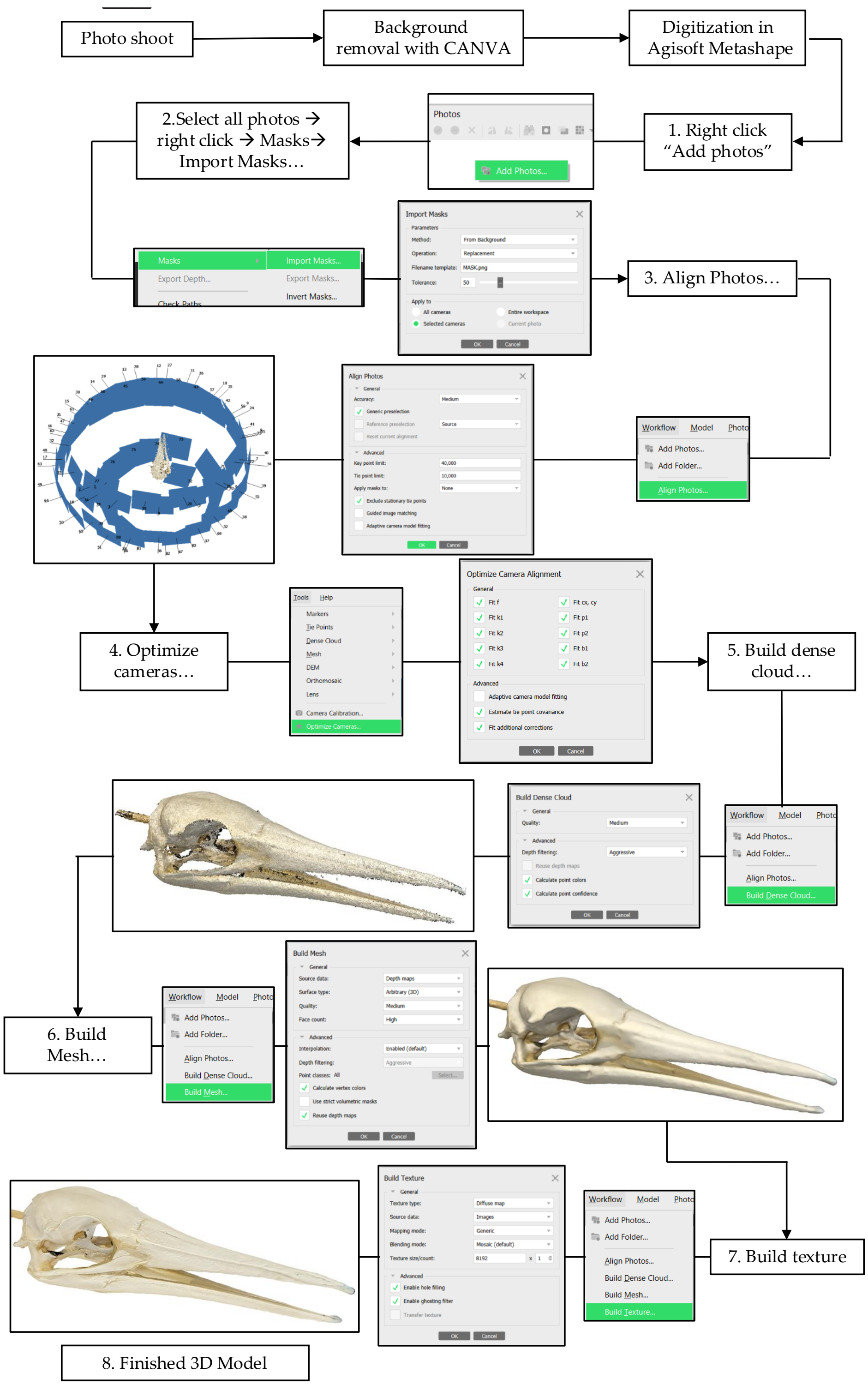

S3 Appendix. A workflow was designed to obtain 3D digitizations of an adult skull of the Peruvian booby (*Sula variegata*, 270 mm height × 60 mm diameter) (Tschudi, 1843). The specimen was complete, with an immobile mandible and without a keratinized covering. This procedure was performed using the AutoScan3D photogrammetry device and micro-CT scanning with a Bruker SkyScan 1278 system (version 1.0.5) to validate the designed device. In addition to the skull, we also digitized a vertebra of the common carp (*Cyprinus carpio*) and a cervical vertebra of the southern pudu (*Pudu puda*), in order to evaluate performance across different vertebrate structures with varied size, surface texture, and anatomical complexity

Detailed Workflow and Assembly Guidance

Overview

This appendix provides a complete and replicable workflow for assembling, calibrating, and operating the AutoScan3D system. The revised version includes:
(i) a full electronic circuit diagram with pin-level labeling,
(ii) a detailed description of potential fabrication pitfalls and solutions, and
(iii) precise, step-by-step operational instructions for users without engineering experience.

1. Electronic Circuit and Pin Connections

A complete wiring schematic has been included as Figure S5 (KiCAD-based diagram). This diagram shows all pin assignments between the Arduino UNO and peripheral components:

- NEMA 17 stepper motor (vertical motion): connected to Pololu A4988 driver → Arduino pins D4 (STEP), D5 (DIR).
- 28BYJ-48 stepper motor (rotation): connected to ULN2003 driver → Arduino pins D8–D11 (IN1–IN4).
- MG995 servo motor (angle adjustment): signal to pin D12.
- MG90 servo motor (Bluetooth shutter trigger): signal to pin D13.
- Power supply: 12 V, 2 A adapter → Pololu VMOT (+), capacitor (100 μF) across VMOT and GND; common ground shared between drivers and Arduino 5 V line.

All connections, wire colors, and power lines are clearly labeled in the schematic for visual assembly.

2. Fabrication Pitfalls and Troubleshooting

Common pitfalls encountered during prototype construction and their solutions include:

Problem	Cause	Solution
**Stepper motor does not rotate**	Wrong driver orientation or missing ground link	Verify A4988 orientation and ensure shared GND between Arduino and driver
**Servo jitter during operation**	Power supply below 12V / <2A	Use stabilized 12V, 2A source or add 100 μF capacitor near servos
**Bluetooth shutter fails**	Delay between command and phone app	Adjust delay (1000) in takePhotoSet() to 1500–2000 ms
**Camera loses focus**	AE/AF lock not activated	Activate "AE/AF lock" before capture sequence
**Uneven lighting**	Lamp too close or diffuser misaligned	Maintain 45° angle and 50 cm distance from diffuser

3. Defining Camera Positions and System Calibration

To define the five vertical camera positions, users should:

1. Measure the total specimen height.
2. Set the value of objectLength in the Arduino code (e.g., objectLength = 200; // mm).
3. The system will automatically divide this distance into five equal vertical intervals using the internal function moveVerticalRail(mm, directionUp), which calculates the number of steps according to stepsPerMm = 143.0.
4. The vertical range corresponds to 20%, 40%, 60%, 80%, and 100% of the total travel, automatically stopping at each level.

The previous instruction "Define five vertical camera positions with 2× zoom" has now been replaced by a clear, programmable explanation consistent with the code in Appendix 2.

4. Step-by-Step Operation Workflow

1. Connect the Arduino UNO via USB and upload the final code (Appendix 2).
2. Pair the smartphone with a Bluetooth shutter app (e.g., *Camera Remote*).
3. Configure camera: resolution 12 MP, 2× zoom, AE/AF lock active.
4. Place the object on the rotation module and ensure the background is uniform.
5. Run the program: the system automatically performs three full capture sets (downward, frontal, and upward).
6. Transfer all images to the computer; perform background removal in CANVA or GIMP using transparency masks.
7. Process images in Agisoft Metashape (or Meshroom) following the workflow illustrated in Fig. S6:
    - Import images → Apply masks → Align photos (High accuracy, Key Point limit 60,000).
    - Optimize cameras → Build mesh (Depth maps, High quality, Moderate filtering).
    - Build texture → Export .OBJ → Inspect in Blender or GOM Inspect.

5. User Notes and Accessibility

- All files necessary for replication—including KiCAD schematic, STL templates, and editable Arduino code—are hosted in Zenodo repository DOI: [https://doi.org/10.5281/zenodo.15644408].
- The entire documentation is available under the MIT Open Hardware License, ensuring that users can freely modify, adapt, and share improvements.
