# Supplementary material for: AutoScan3D: A low-cost, portable photogrammetry system for automated 3D digitization of anatomical specimens
Source: PLoS One. 2025 Nov 19;20(11):e0336996. doi: 10.1371/journal.pone.0336996 (PMC12629479; doi:10.1371/journal.pone.0336996)
Supplement: S4 Appendix — This appendix offers a comprehensive protocol for assembling, configuring, and operating AutoScan3D—a low-cost photogrammetry device for automated 3D digitization of anatomical specimens. It includes instructions for construction, circuit configuration, Arduino UNO programming, photographic setup, and image processing. (PDF) [file pone.0336996.s004.pdf]

Appendix 4

Step-by-Step: AutoScan3D Construction and Operation Guide

Overview

This guide provides a detailed step-by-step protocol for constructing, configuring, and operating AutoScan3D, a low-cost photogrammetry device designed for 3D digitization of anatomical specimens. The system integrates a smartphone camera, stepper motors, and an Arduino UNO for automated image capture. Total hardware cost is approximately $90 USD.

Materials

- **Hardware components (see Table 1):**
  - Arduino UNO board
  - Nema 17 stepper motor (camera positioning)
  - 28BYJ-48 stepper motor (object rotation)
  - MG90 and MG995 servo motors (shutter trigger, positioning)
  - Pololu A4988 and ULN2003 motor drivers
  - 5/16" threaded rod, telescopic slide rail (std h-35 400 mm), MDF (9 mm thick)
  - Smartphone (e.g., Samsung Galaxy A15 5G, width ≤80 mm, ≥12 MP camera, Bluetooth)
  - 12V, 2A power adapter, Dupont cables, 100 μF capacitor
- **Tools:** Screwdrivers, pliers, cyanoacrylate adhesive, *matte* black acrylic paint.
- **Software:** Arduino IDE 2.3.3 (free), Agisoft Metashape Professional ($3,499 USD) or open-source alternatives (e.g., Meshroom).

Construction Steps

1. Camera Positioning Module

- Build a circuit housing (MDF, 9 mm thick) with internal dimensions of 120 × 80 × 60 mm. Include openings for the Nema 17 motor shaft (14 mm) and cable routing.
- Mount a telescopic slide rail (std h-35 400 mm) perpendicular to the base using MDF joints and M6/M4 fasteners.
- Attach a 5/16" threaded rod to the Nema 17 motor via a modified expansion plug (5 mm bore), securing it with double nuts and cyanoacrylate adhesive.
- Integrate an MG995 servo motor for fine stability adjustment and position locking.
- All joints should be bonded with cyanoacrylate adhesive for reinforcement.

2. Rotation and Photo Capture Module

- Construct a box using MDF (9 mm thick) measuring 100 × 100 × 60 mm. The upper face includes a 14 mm central aperture for the telescopic rod.
- Mount a 28BYJ-48 stepper motor inside the box, connected to a cylindrical plastic piece with an embedded M4 nut, which joins to the telescopic rod (repurposed from a selfie stick).
- Install an MG90 servo motor with a plastic arm to trigger the smartphone's Bluetooth shutter.
- Paint all outer surfaces with matte black paint to reduce specular reflections and improve image uniformity during photogrammetry. Glossy finishes were previously tested but produced unwanted highlights and angle-dependent contrast artifacts.
- The threaded rod, cables, and telescopic rod remain unpainted.
- Use a 1-meter Dupont cable strip to connect the module to the control housing.

3. Electronic Circuit

- Connect the 28BYJ-48 motor to the ULN2003 driver (IN1–IN4 to Arduino pins 8–11).
- Wire the Nema 17 motor to the Pololu A4988 driver (pins 2B, 2A, 1A, 1B) with a 100 μF capacitor and 12V, 2A power jack.
- Connect the MG90 servo (pin 13) and MG995 servo (pin 12) to Arduino UNO (GND, +5V).
- Link all modules with a 1 m Dupont cable strip to maintain modular assembly.

4. Smartphone Holder

- Attach a universal holder (maximum opening 80 mm) to the vertical rail, compatible with smartphones ≤80 mm in width.
- Ensure the device supports Bluetooth shutter control, manual focus, and a ≥12 MP camera (e.g., Samsung Galaxy A15 5G or iPhone 8).

Configuration Steps

1. Arduino Programming

- Download Arduino IDE 2.3.3 from https://www.arduino.cc/en/software.
- Upload the AutoScan3D code (Appendix 2) to the Arduino UNO, defining:
    - `int numberofsteps = 24000;  // Nema 17 motor vertical range`
    - `motor.set(55);              // MG995 servo initial position`
    - `for(int i = 0; i < 32; i++); // 28BYJ-48 motor (32° steps)`
    - `motor.set(45);              // MG90 servo trigger`
- Test individual motor movements on a **protoboard (MB-102, 830 points)** before final assembly.

2. Smartphone Setup

- Install a Bluetooth shutter app (*e.g.*, Camera Remote).
- Configure manual camera settings: focus locked, 2× zoom, high resolution (50 MP).
- Align the smartphone camera with the object's center axis.

3. Photographic Environment

- Prepare a 2 × 1.6 m matte black backdrop, positioned parallel to a window for diffused daylight.
- Place a 15W LED lamp (E27) at a 45° angle, 1.5 m from the object, with a 110 cm translucent diffuser 50 cm from the light source.
- Mount the specimen (*e.g.*, skull) on a bamboo rod fixed into a cork base and attached to the telescopic rod.

Operation Steps

1. Image Capture

- Define five vertical positions using the Nema 17 motor.
- Capture five sets of 16 photographs (≈83 total) at 22.5° rotation intervals using the 28BYJ-48 motor.
- The MG995 servo adjusts the camera tilt, and the MG90 servo triggers the Bluetooth shutter.

2. Image Processing

- Transfer images to a computer and organize them in folders.
- Remove backgrounds using CANVA (www.canva.com) and export as PNG with a MASK.png file.
- In Agisoft Metashape Professional 1.8.4:
  - Align photos (Accuracy: *High*, Key Point Limit: 60,000, Tie Point Limit: 30,000).
  - Optimize cameras, build mesh from Depth Maps (High quality), and apply texture (Mapping Mode: Generic).
  - Export the model as a .OBJ file.

3. Validation

- Open the exported model in Blender 4.2 to verify the vertex count.
- Compare AutoScan3D models with micro-CT references using GOM Inspect to assess surface deviations.

Additional Notes

- Dimensions (example): Circuit housing – 120 × 80 × 60 mm; Rotation box – 100 × 100 × 60 mm. These can be modified ±10 mm depending on local materials.
- Paint Recommendation: Use matte black acrylic paint (<10 gloss units @60°) to minimize reflections and improve photogrammetric consistency.
- Total assembly weight: ~441 g.
- Cost breakdown: Hardware $90 USD; optional software $3,499 USD (Agisoft). Use Meshroom for a free alternative.
- Data availability: All code, STL files, and design templates are openly available at Zenodo (https://doi.org/10.5281/zenodo.15644408).

Software Alternatives:
While Agisoft Metashape (Professional 1.8.4) was used in this study, comparable open-source software such as Meshroom (AliceVision framework) can serve as a free alternative for 3D reconstruction. Several comparative analyses report that Meshroom produces models of similar geometric accuracy and surface detail to Metashape for small and medium-sized objects, with differences typically below 3 mm in mean surface deviation, although reconstruction time is longer (Olowinsky et al., 2022; Pérez et al., 2023; Aguilar et al., 2024). Thus, Meshroom provides a viable open-source option for educational and low-budget photogrammetry applications.
