# Supplementary material for: AutoScan3D: A low-cost, portable photogrammetry system for automated 3D digitization of anatomical specimens
Source: PLoS One. 2025 Nov 19;20(11):e0336996. doi: 10.1371/journal.pone.0336996 (PMC12629479; doi:10.1371/journal.pone.0336996)
Supplement: S5 Appendix — This appendix presents the complete electronic circuit model of the AutoScan3D system, illustrating the integration between mechanical modules and their electronic components. The diagram, designed in Fritzing, details all connections among actuators, drivers, and the Arduino UNO control board. (PDF) [file pone.0336996.s005.pdf]

S5 Appendix

Electronic circuit model and wiring scheme of the AutoScan3D device. This appendix presents the complete electronic circuit model of the AutoScan3D system, illustrating the integration between the mechanical modules and their electronic components. The diagram was designed in **fritzing** and details the connections among all actuators, drivers, and the Arduino UNO control board.

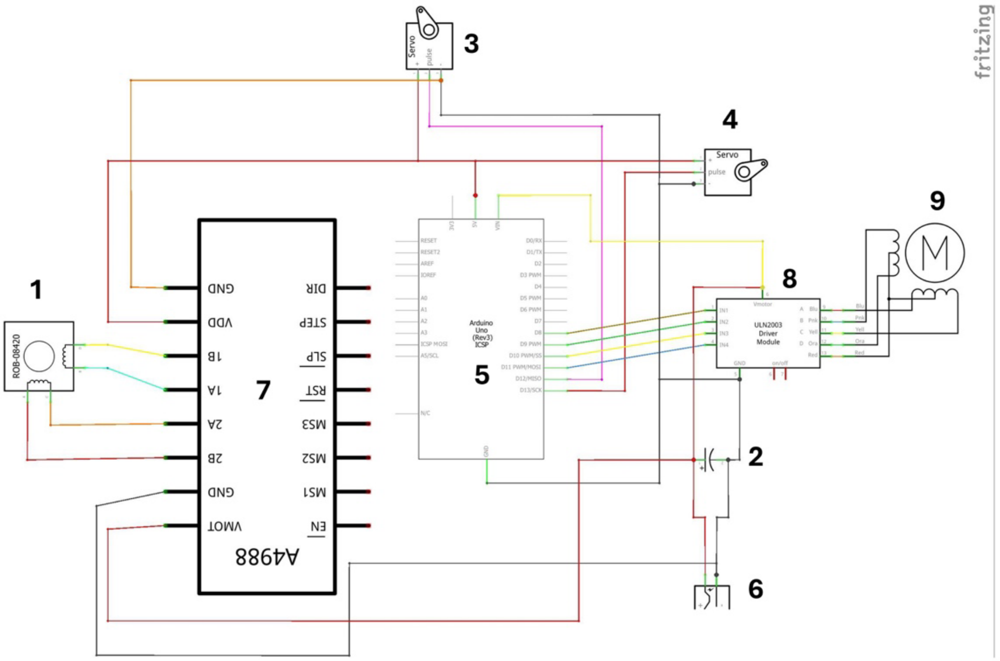

Fig 1. (A) Circuit scheme of AutoScan3D device. (1) MG90 servo motor triggering the smartphone's Bluetooth shutter and a 28BYJ-48 stepper motor with an M4 nut, connected to (2) a telescopic rod repurposed from a selfie stick, enabling controlled object rotation. The assembly is coated in black paint except for the 5/16" threaded rod, cables, and telescopic rod. The camera positioning control module consists of (3) a circuit housing with openings for the Nema 17 motor shaft (14 mm) and cable routing. Vertical camera movement is managed by (4) a "std h-35 400 mm" telescopic slide rail with linear bearings, secured with MDF joints and fasteners. Elevation control is achieved via a 5/16" threaded rod, coupled to the Nema 17 motor through a modified expansion plug and guided by 5/16" nuts. (5) An MG995 servo motor enhances stability and precise positioning. (B) Electronic circuit diagram illustrating the interconnection of key components, including (1) Nema 17 stepper motor, (2) 100 μF capacitor, (3) MG90 servo motor, (4) MG995 servo motor, (5) Arduino UNO board, (6) 5.5x2.1 mm female jack for power supply, (7) Pololu A4988 driver, (8) ULN2003 driver, and (9) 28BYJ-48 stepper motor. This system facilitates high-precision, multi-angle image capture for anatomical and morphological studies.
